# Supplementary material for: Mechanistic Modelling Identifies and Addresses the Risks of Empiric Concentration-Guided Sorafenib Dosing
Source: Pharmaceuticals (Basel). 2021 Apr 21;14(5):389. doi: 10.3390/ph14050389 (PMC8143107; doi:10.3390/ph14050389)
Supplement: Supplementary file 1 [file pharmaceuticals-14-00389-s001.zip › pharmaceuticals-1159675-supplementary.pdf]

SUPPLEMENTARY DATA

Table S1. Verification of the impact of drug interactions on sorafenib exposure.

Trials	Age (median [range])	Female (%)	Dosing regimen	Trial	C_{max} (mg/L)	AUC (mg/L.hr)
Strumberg D, Clark JW, Awada A et al. [3]	60 [18 - 75]	36 %	Single dose 100 mg (n = 3)	Observe	2.69	83.80
				Simulated	0.55	6.93
				Ratio	4.89	12.09
			Single dose 400 mg (n = 3)	Observe	3.42	107.00
				Simulated	2.21	27.72
				Ratio	1.55	3.86
			Multiple dose 100 mg (n = 3)	Observe	2.31	23.80
				Simulated	1.42	14.60
				Ratio	1.63	1.63
			Multiple dose 200 mg (n = 3)	Observe	2.84	16.10
				Simulated	2.84	29.20
				Ratio	1.00	0.55
				Observe	9.35	71.70

			Multiple dose 400 mg (n = 5)	Simulated	5.68	58.39
				Ratio	1.65	1.23
			Multiple dose 600 mg (n = 8)	Observe	9.81	79.00
				Simulated	8.53	87.59
				Ratio	1.15	0.90
Minami H, Kawada K, Ebi H et al. [36]	63 [32 - 73]	32 %	Single dose 100 mg (n = 3)	Observe	0.43	9.40
				Simulated	0.53	6.40
				Ratio	0.81	1.47
			Single dose 200 mg (n = 15)	Observe	0.74	24.30
				Simulated	1.12	13.42
				Ratio	0.66	1.81
			Single dose 400 mg (n = 6)	Observe	1.21	35.4
				Simulated	2.11	25.51
				Ratio	0.57	1.39
			Single dose 600 mg (n = 7)	Observe	1.41	40.50
				Simulated	3.13	37.21
				Ratio	0.45	1.09

Awada A, Hendlisz A, Gil T et al. [4]	58 [42 - 79]	43 %	200 mg BID mg (n = 3) Day 1	Observe	3.63	24.90
				Simulated	1.57	8.80
				Ratio	2.31	2.83
			400 mg BID mg (n = 9) Day 1	Observe	3.04	24
				Simulated	3.18	28.17
				Ratio	0.96	0.85
			600 mg BID mg (n = 12) Day 1	Observe	4.56	30.40
				Simulated	4.81	42.65
				Ratio	0.95	0.71
			200 mg BID mg (n = 3) Day 7	Observe	9.01	83.40
				Simulated	3.48	36.20
				Ratio	2.59	2.30
			400 mg BID mg (n = 9) Day 7	Observe	9.90	82.70
				Simulated	6.61	68.79
				Ratio	1.50	1.20
			600 mg BID mg (n = 12)	Observe	11.50	94.80
				Simulated	10.10	109.72

			Day 7	Ratio	1.14	0.86
			200 mg BID mg (n = 3) Day 21	Observe	6.33	50.50
				Simulated	4.05	45.57
				Ratio	1.56	1.11
			400 mg BID (n = 5) Day 21	Observe	10	76.50
				Simulated	7.8	87.33
				Ratio	1.28	0.88
			600 mg BID (n = 12) Day 21	Observe	9.24	77
				Simulated	11.36	125.74
				Ratio	0.81	0.61

Table S2. Summary of physiological and molecular characteristic considered in regression analyses.

Parameter	Mean	Standard Deviation	Range	
			5th centile	95th centile
Sorafenib Exposure				
Steady state C_{max} (mg/L)	8.92	3.35	4.58	15.3
Steady state AUC (mg/L.hr)	99.2	39.6	48.7	176
Physiological Characteristics				
Female (%)	50			
Age (years)	40.4	9.05	22.6	49.6
Weight (kg)	74.6	14.8	53.0	102
Height (cm)	170	9.38	155	184
BMI (kg/m^2)	25.9	4.28	19.8	33.4
Cardiac output (L/hr)	312	38.0	254	381
Haematocrit (%)	38.0	4.86	30.7	46.5
Albumin (g/L)	39.5	7.26	28.9	53.0
GFR (mL/min/1.73m^2)	109	24.2	53.3	217
Liver CYP abundance (pmol P450)				
CYP3A4	8523459	4988135	1378085	44856000
Liver UGT abundance (pmol protein)				
UGT1A9	1773543	873240	4051	5530162
Intestinal CYP abundance (pmol P450)				
CYP3A4	64549	38872	9653	322094
Intestinal UGT abundance (pmol protein)				
UGT1A9	16689	11925	1621	63907

Abbreviations: BMI = body mass index, GFR = glomerular filtration rate, CYP = cytochrome P450, UGT = Uridine 5'-diphospho-glucuronosyltransferase.

Table S3. Logistic regression analysis of therapeutic C_{max} threshold of > 5.5926 mg/L.

Parameter	OR	95% CI	Regression *p* value	C-statistic
Physiological Characteristics				
Sex (Male/Female)	1.06	0.71 to 1.58	0.792	0.51
Age# (years)	1.23	1.00 to 1.52	0.050	0.56
Weight# (kg)	0.53	0.46 to 0.61	<0.001	0.76
Height# (cm)	0.65	0.53 to 0.81	<0.001	0.61
Body Surface Area (m^2)	0.02	0.01 to 0.04	<0.001	0.74
BMI (kg/m^2)	0.82	0.79 to 0.86	<0.001	0.73
Albumin (g/L)	1.07	1.04 to 1.10	<0.001	0.62
Creatinine (µmol/L)	1.00	0.99 to 1.02	0.636	0.53
GFR# (mL/min/1.73m^2)	0.13	0.06 to 0.29	<0.001	0.66
CYP3A4 abundance (µmol P450)				
Intestinal	0.16	0.00 to 22.4	0.469	0.5
Hepatic	0.57	0.52 to 0.63	<0.001	0.96
UGT1A9 abundance (µmol P450)				

Intestinal[#]	0.16	0.84 to 1.17	0.903	0.49
Hepatic	0.49	0.40 to 0.61	<0.001	0.69

[#] Age per ten years incremental, weight per 10 kg incremental, height per 10 cm incremental, GFR per 100 ml incremental, intestinal UGT1A9 per 10,000 pmol incremental

OR = odd ratio, CI = confidence interval, BMI = Body Mass Index, GFR = Glomerular filtration rate
